# Supplementary material for: Automatic Cardiopulmonary Endurance Assessment: A Machine Learning Approach Based on GA-XGBOOST
Source: Diagnostics (Basel). 2022 Oct 19;12(10):2538. doi: 10.3390/diagnostics12102538 (PMC9600669; doi:10.3390/diagnostics12102538)
Supplement: Supplementary file 1 [file diagnostics-12-02538-s001.zip › diagnostics-1940558-supplementary.pdf]

1. C-OWA(MATLAB)

```
clc
clear
close all
x=[9 8 8 8 9
    9 7 8 7 8
    8 7 7 7 7
    8 8 8 7 8
    8 6 7 6 8
    9 7 8 6 8
    8 7 7 6 8
    8 6 7 6 7
    ]; %cardiovascular capacity
%row heading is the expert number, and the column heading is the indicator name
x=sort(x,'descend');   %descending order
[m,n]=size(x);
for i=1:m
    c(i)=nchoosek(m-1,i-1);    %calculate number of combinations
end
a=c' ./ (2^(m-1)) ;   %weight vector

for j=1:n
    wj(:,j)=a.*x(:,j);    %absolute weight
end
wj=sum(wj);
w=wj./sum(wj)    %relative weight
%result

% x=[8 8 7 8
%     8 7 6 7
%     9 9 8 9
%     8 8 7 8
%     8 7 7 8
%     9 8 7 8
%     8 8 7 8
%     8 6 5 7
%     ];  %Respiratory exchange capacity
% x=[8 9 6 7 8
%     9 9 6 6 8
%     8 9 6 7 8
%     8 8 7 7 7
%     9 9 6 8 8
```

```
%       8 9 7 7 8
%       7 9 6 7 7

%       8 9 7 7 7] %metabolic ability
```

```
w =

    0.2213    0.1883    0.2017    0.1750    0.2137

fx >>
```

2. THE ENTROPY WEIGHT METHOD (MATLAB)

```
data=xlsread('D:\Desktop\shangquan.xlsx')
data=data(:,3:end) %Only the indicator data is taken
data1=data;
index=[3,4];
for i=1:length(index)
  data1(:,index(i))=max(data(:,index(i)))-data(:,index(i));
end
index=[5];
a=90;
for i=1:length(index)
  data1(:,index(i))=1-abs(data(:,index(i))-a)/max(abs(data(:,index(i))-a))
end
data2=mapminmax(data1',0.001,1)
data2=data2';
[m,n]=size(data2);
p=zeros(m,n);
for j=1:n
    p(:,j)=data2(:,j)/sum(data2(:,j))
end
for j=1:n
   E(j)=-1/log(m)*sum(p(:,j).*log(p(:,j)))
end
%calculate weight
w=(1-E)/sum(1-E)
```

```
w1 =

    0.0730    0.0620    0.0660    0.0580    0.0700    0.0820    0.0790    0.0710    0.0810    0.0760    0.0850    0.0590    0.0660    0.0730

w2 =

    0.0840    0.0500    0.0820    0.0350    0.0820    0.1140    0.0440    0.0800    0.0940    0.0490    0.1460    0.0440    0.0380    0.0560

w =

    0.0780    0.0580    0.0730    0.0480    0.0750    0.0960    0.0640    0.0750    0.0870    0.0640    0.1110    0.0530    0.0540    0.0660
```

3. CPETASSESSMENT(PYTHON)

```python
def CPETAssessment(Data):
    import pandas as pd
    import numpy as np
    import math
    from scipy import stats
    Parameters = Data.values.tolist()
    Parameters = np.array(Parameters)
    # data format conversion
    # Power=Parameters[:,1].astype('float32')
    Power = Parameters[:, 2].astype('float32')
    HR = Parameters[:, 3].astype('float32')
    SV = Parameters[:, 4].astype('float32')
    CO = Parameters[:, 5].astype('float32')
    VO2 = Parameters[:, 6].astype('float32')
    VCO2 = Parameters[:, 7].astype('float32')
    VE = Parameters[:, 8].astype('float32')
    BOA = Parameters[:, 9].astype('float32')

    # ventilation equivalent
    EQO2 = VE * 1000. / VO2
    EQO2 = np.array(EQO2)  # print(EQO2.shape)
    EQCO2 = VE * 1000. / VCO2
    EQCO2 = np.array(EQCO2)
    # oxygen pulse
    OP = VO2 / HR
    # RQ
    RQ = VCO2 / VO2
    # Ca_VO2
    Ca_vO2 = VO2 / (HR * SV)

    # new array
    parameters = np.vstack((Parameters[:, 1], Power, HR, SV, CO,
VO2, VCO2, VE, EQO2, EQCO2 , OP, RQ, Ca_vO2,  BOA))
```

```
parameters = np.transpose(parameters)
np.savetxt("parameters.csv", parameters, delimiter=',', fmt='%s')

##拐点检测 AT 和力竭时间点
# anaerobic threshold
rows = len(Parameters)

for i in range(0, rows - 1):
    if (VO2[i] < VCO2[i] and VO2[i + 1] < VCO2[i + 1]):
        time_AT = Parameters[i, 1]
        print('AT 的时间为%s' % time_AT)
        AT = i
        break
# exhaustion time point
for i in range(0, rows - 1):
    if (Power[i + 1] < Power[i]):
        time_MAX = Parameters[i, 1]
        print('力竭的时间为%s' % time_MAX)
        MAX = i
        break

# Physiological characteristic parameters

x1 = HR[AT]
HR_AT=HR[AT]
x1_max = 173
x1_min = 71
# x1=(HR[AT]-x1_min)/(x1_max-x1_min)
x2 = HR[MAX]
x2_max = 198
x2_min = 115
# x2=(HR[MAX]-x2_min)/(x2_max-x2_min)
x3 = SV[AT]
x3_max = 132
x3_min = 8.8
# x3=(SV[AT]-x3_min)/(x3_max-x3_min)
x4 = SV[MAX]
x4_max = 192.1
x4_min = 12.1
# x4=(SV[MAX]-x4_min)/(x4_max-x4_min)
x6 = OP[MAX]
x6_max = 22.6
```

```
    x6_min = 6.8
    # x6=(OP[MAX]-x6_min)/(x6_max-x6_min)
    # Gas exchange function
    x7 = EQO2[AT]
    x7_max = 33.1
    x7_min = 20.2
    # x7=(x7_max-EQO2[AT])/(x7_max-x7_min)
    x8 = EQO2[MAX]
    x8_max = 72.3
    x8_min = 22.5
    # x8=(x8_max-EQO2[MAX])/(x8_max-x8_min)
    x9 = VE[AT]
    x9_max = 82.8
    x9_min = 18.3
    # x9=(VE[AT]-x9_min)/(x9_max-x9_min)
    x10 = VE[MAX]
    x10_max = 168.6
    x10_min = 28.4
    # x10=(VE[MAX]-x10_min)/(x10_max-x10_min)
    # Metabolic functions
    x11 = VO2[AT]
    x11_max = 39.4
    x11_min = 14.06
    # x11=(VO2[AT]-x11_min)/(x11_max-x11_min)
    x12 = VO2[MAX]
    x12_max = 61.02
    x12_min = 21.68
    # x12=(VO2[MAX]-x12_min)/(x12_max-x12_min)
    x14 = RQ[MAX]
    x14_max = 1.47
    x14_min = 0.97
    # x14=(RQ[MAX]-x14_min)/(x14_max-x14_min)
    x15 = Ca_vO2[AT]
    x15_max = 0.977
    x15_min = 0.105
    # x15=(Ca_vO2[AT]-x15_min)/(x15_max-x15_min)
    x16 = Ca_vO2[MAX]
    x16_max = 0.880
    x16_min = 0.0916
    # x16=(Ca_vO2[MAX]-x16_min)/(x16_max-x16_min)

    #  MroScore
    # Define positive and negative ideal solutions
```

```python
    # Define the initial evaluation matrix
    matrix = np.zeros((3, 14))
    matrix[0, :] = [x1, x2, x3, x4, x6, x7, x8, x9, x10, x11, x12, x14, x15, x16]
    matrix[1, :] = [x1_max, x2_max, x3_max, x4_max, x6_max, x7_max, x8_max, x9_max, x10_max, x11_max, x12_max, x14_max,
                    x15_max, x16_max]
    matrix[2, :] = [x1_min, x2_min, x3_min, x4_min, x6_min, x7_min, x8_min, x9_min, x10_min, x11_min, x12_min, x14_min,
                    x15_min, x16_min]
    # integer ideal solution
    D = np.zeros((2, 14))
    s1 = 0
    s2 = 0
    Weight = [0.078, 0.058, 0.073, 0.048, 0.075, 0.096, 0.064, 0.075, 0.087, 0.064, 0.111, 0.053, 0.054, 0.066]
    for i in range(0, 13):
        if (i != 7 and i != 8):
            D[0, i] = Weight[i] * ((matrix[0, i] - matrix[1, i]) / (matrix[1, i] - matrix[2, i]))
            s1 = s1 + pow(D[0, i], 2)
        else:
            D[0, i] = Weight[i] * ((matrix[2, i] - matrix[0, i]) / (matrix[1, i] - matrix[2, i]))
            s1 = s1 + pow(D[0, i], 2)
    s1 = math.sqrt(s1)
    for i in range(0, 13):
        if (i != 7 and i != 8):
            D[1, i] = Weight[i] * ((matrix[0, i] - matrix[2, i]) / (matrix[1, i] - matrix[2, i]))
            s2 = s2 + pow(D[1, i], 2)
        else:
            D[1, i] = Weight[i] * ((matrix[1, i] - matrix[0, i]) / (matrix[1, i] - matrix[2, i]))
            s2 = s2 + pow(D[1, i], 2)
    s2 = math.sqrt(s2)
    MroScore = 70.855 * s2 / (s1 + s2) + 46.615
    MroScore = round(MroScore, 1)
    print('心肺耐力综合得分为%10.1f' % MroScore)


    #cardiovascular capacity Score1
    Weight1 = [0.235, 0.175, 0.220, 0.144, 0.226]
```

```python
    D = np.zeros((2, 5))
    s1 = 0
    s2 = 0
    for i in range(0, 4):
        D[0, i] = Weight[i] * ((matrix[0, i] - matrix[1, i]) / (matrix[1, i] - matrix[2, i]))
        s1 = s1 + pow(D[0, i], 2)
    s1 = math.sqrt(s1)
    for i in range(0, 5):
        D[1, i] = Weight[i] * ((matrix[0, i] - matrix[2, i]) / (matrix[1, i] - matrix[2, i]))
        s2 = s2 + pow(D[1, i], 2)
    s2 = math.sqrt(s2)
    Score1 = 70.855 * s2 / (s1 + s2) + 40.615
    print(s2 / (s1 + s2) )
    Score1 = round(Score1, 1)
    print('心血管能力得分为%10.1f' % Score1)

    # 呼吸能力  Score2
    Weight1 = [0.298, 0.198, 0.223, 0.270]
    D = np.zeros((2, 4))
    s1 = 0
    s2 = 0
    for i in range(0, 3):
        D[0, i] = Weight[i] * ((matrix[0, i + 5] - matrix[1, i + 5]) / (matrix[1, i + 5] - matrix[2, i + 5]))
        s1 = s1 + pow(D[0, i], 2)
    s1 = math.sqrt(s1)
    for i in range(0, 4):
        D[1, i] = Weight[i] * ((matrix[0, i + 5] - matrix[2, i + 5]) / (matrix[1, i + 5] - matrix[2, i + 5]))
        s2 = s2 + pow(D[1, i], 2)
    s2 = math.sqrt(s2)
    Score2 = 69.855 * s2 / (s1 + s2) + 48.615
    Score2 = round(Score2, 1)
    print('呼吸能力得分为%10.1f' % Score2)

    # metabolic capacity  Score3
    Weight1 = [0.184, 0.319, 0.152, 0.155, 0.190]
    D = np.zeros((2, 5))
    s1 = 0
    s2 = 0
    for i in range(0, 4):
        D[0, i] = Weight[i] * ((matrix[0, i + 9] - matrix[1, i +
```

```python
9]) / (matrix[1, i + 9] - matrix[2, i + 9]))
        s1 = s1 + pow(D[0, i], 2)
    s1 = math.sqrt(s1)
    for i in range(0, 5):
        D[1, i] = Weight[i] * ((matrix[0, i + 9] - matrix[2, i + 9]) / (matrix[1, i + 9] - matrix[2, i + 9]))
        s2 = s2 + pow(D[1, i], 2)
    s2 = math.sqrt(s2)
    Score3 = 70.855 * s2 / (s1 + s2) + 44.615
    Score3 = round(Score3, 1)
    print('代谢能力得分为%10.1f' % Score3)
    result=[time_AT,time_MAX,MroScore,Score1,Score2,Score3,HR_AT]
    return result
```

```python
import pandas as pd
import CPETAssessment
Data = pd.read_csv(r'D:\Users\LiuQi\Desktop\data_lq.csv')
result=CPETAssessment.CPETAssessment(Data)
print(result)
```

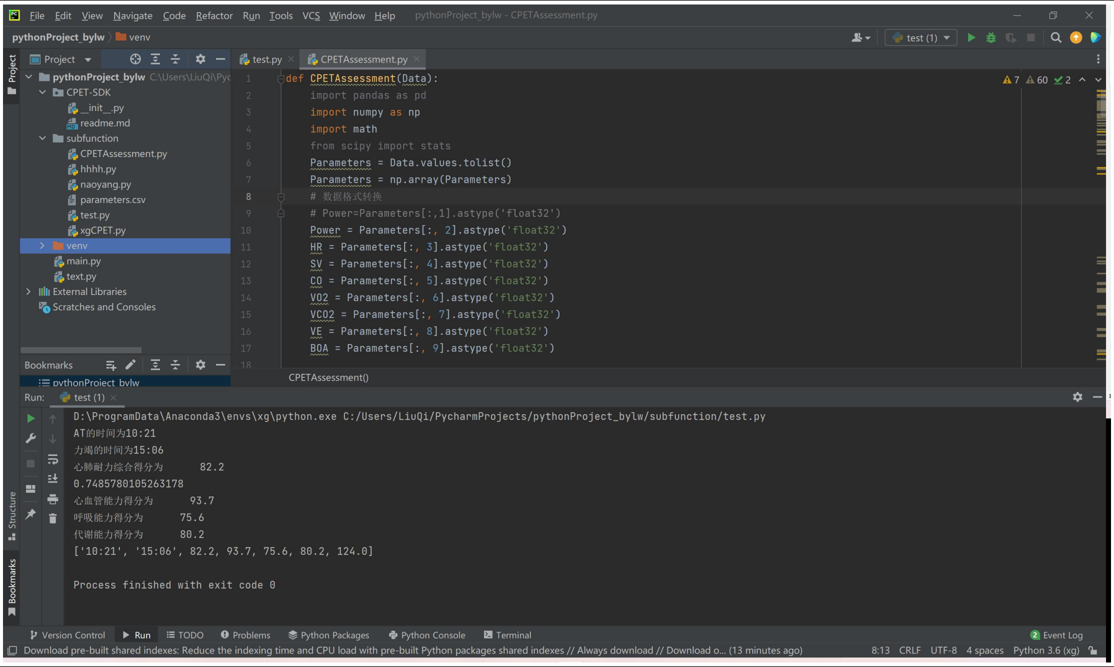

4. GA_XGBOOST(PYTHON)

```python
# coding=utf-8
```

```
from __future__ import division
import numpy as np
import pandas as pd
import random
import math
from sklearn import metrics
from sklearn.model_selection import train_test_split
import xgboost as xgb
from random import randint

# from xgboost.sklearn import XGBClassifiers

# generations = 400   # Generations 100
pop_size = 500  # Population quantity  500
# max_value = 10      # The maximum value allowed in a gene (to prevent the number of discrete variables from reaching a power of two, limit the maximum value, not used here)
chrom_length = 15  # Length of chromosome
pc = 0.6  # Crossover probability
pm = 0.01  # Mutation probability
results = []  # Optimal solution（auc 最高值，n_estimators, max_depth）
fit_value = []  # 个体适应度
fit_mean = []  # 平均适应度
# pop = [[0, 1, 0, 0, 0, 1, 0, 1, 0, 1, 0, 1, 0, 1, 0] for i in range(pop_size)] # Initializes the initial sequence of genes of all individuals in the population

random_seed = 20
cons_value = 0.19 / 31  # (0.20-0.01）/ (32 - 1)

'''The parameters to debug are: （Reference: http://xgboost.readthedocs.io/en/latest/parameter.html）
   tree_num: Number of base trees   ----------------（parameters to debug）
   eta: learning_rate, default value 0.3, range of [0,1]  ----------------（parameters to debug）
   max_depth: default value 6   ----------------（parameters to debug）

   Three of the above parameters should be adjusted, and the rest can be adjusted by ideal values
'''
```

```python


def xgboostModel(tree_num, eta, max_depth, random_seed):
    train_xy = loadFile("../../Data/train-gao.csv")
    train_xy = train_xy.drop('ID', axis=1)  #  Delete the ID of the training set
    # Divide the training set into a ratio of 8:2 (training set to validation set ratio)
    train, val = train_test_split(
        train_xy, test_size=0.2, random_state=80)

    train_y = train.Kind
    train_x = train.drop('Kind', axis=1)
    dtrain = xgb.DMatrix(train_x, label=train_y)

    val_y = val.Kind
    val_x = val.drop('Kind', axis=1)
    dval = xgb.DMatrix(val_x)

    params = {
        'booster': 'gbtree',  # gbtree used
        'objective': 'binary:logistic',
        'early_stopping_rounds': 100,
        # 'scale_pos_weight': 0.13,  # Positive sample weight
        'eval_metric': 'auc',
        'eta': eta,  # 0.02
        'max_depth': max_depth,  # 8
        'gamma': 0.1,
        'subsample': 0.8,
        'colsample_bytree': 0.8,
        'lambda': 550,
        'alpha': 19,
        'seed': randint(1, 10000),
        'nthread': 3,
        'silent': 1
    }
    model = xgb.train(params, dtrain, num_boost_round=tree_num)
    predict_y = model.predict(dval, ntree_limit=model.best_ntree_limit)
    roc_auc = metrics.roc_auc_score(val_y, predict_y)
    return roc_auc


```

```
def loadFile(filePath):
    fileData = pd.read_csv(filePath)
    return fileData


# Step 1 : Encode the parameters (used to initialize the gene sequence, can be selected to initialize the gene sequence, omitted in this function)
def geneEncoding(pop_size, chrom_length):
    pop = [[]]
    for i in range(pop_size):
        temp = []
        for j in range(chrom_length):
            temp.append(random.randint(0, 1))
        pop.append(temp)
    return pop[1:]


# Step 2 : Calculate the individual objective function value
def cal_obj_value(pop):
    objvalue = []
    variable = decodechrom(pop)
    for i in range(len(variable)):
        tempVar = variable[i]

        tree_num_value = (tempVar[0] + 1) * 10
        eta_value = 0.01 + tempVar[1] * cons_value
        max_depth_value = 3 + tempVar[2]
        min_child_weight_value = 1 + tempVar[3]

        aucValue = xgboostModel(tree_num_value, eta_value, max_depth_value, random_seed)
        objvalue.append(aucValue)
    return objvalue  # Objective function value objvalue[m] corresponds to Individual genes pop[m]


# Decode each individual  and split into individual variables, return tree_num（4）、eta（5）、max_depth（3）
def decodechrom(pop):
    variable = []
    for i in range(len(pop)):
        res = []
```

```

        # Calculate the first variable value， 0101->10(reverse)
        temp1 = pop[i][0:4]
        v1 = 0
        for i1 in range(4):
            v1 += temp1[i1] * (math.pow(2, i1))
        res.append(int(v1))

        # Calculate the second variable value
        temp2 = pop[i][4:9]
        v2 = 0
        for i2 in range(5):
            v2 += temp2[i2] * (math.pow(2, i2))
        res.append(int(v2))

        # Calculate the third variable value
        temp3 = pop[i][9:12]
        v3 = 0
        for i3 in range(3):
            v3 += temp3[i3] * (math.pow(2, i3))
        res.append(int(v3))

        variable.append(res)
    return variable


# Step 3: Calculate individual fitness value (calculate maximum value, eliminate negative value)
def calfitvalue(obj_value):
    fit_value = []
    temp = 0.0
    Cmin = 0
    for i in range(len(obj_value)):
        if (obj_value[i] + Cmin > 0):
            temp = Cmin + obj_value[i]
        else:
            temp = 0.0
        fit_value.append(temp)
    return fit_value


# Step 4: Find the maximum value of the fitness function, and the corresponding individual
```

```
def best(pop, fit_value):
    best_individual = pop[0]
    best_fit = fit_value[0]
    for i in range(1, len(pop)):
        if (fit_value[i] > best_fit):
            best_fit = fit_value[i]
            best_individual = pop[i]
    return [best_individual, best_fit]


# Step 5: Each time evolve, record the best results (convert binary to decimal)
def b2d(best_individual):
    # Calculate the first variable value
    temp1 = best_individual[0:4]
    v1 = 0
    for i1 in range(4):
        v1 += temp1[i1] * (math.pow(2, i1))
    v1 = (v1 + 1) * 10

    # Calculate the second variable value
    temp2 = best_individual[4:9]
    v2 = 0
    for i2 in range(5):
        v2 += temp2[i2] * (math.pow(2, i2))
    v2 = 0.01 + v2 * cons_value

    # Calculate the third variable value
    temp3 = best_individual[9:12]
    v3 = 0
    for i3 in range(3):
        v3 += temp3[i3] * (math.pow(2, i3))
    v3 = 3 + v3

    return int(v1), float(v2), int(v3)

# Step 6: Natural selection (roulette algorithm)
def selection(pop, fit_value):
    # Calculate the probability of each adaptation value
    new_fit_value = []
    total_fit = sum(fit_value)
    for i in range(len(fit_value)):
        new_fit_value.append(fit_value[i] / total_fit)
```

```
    # The cumulative probability of each adaptation
    cumsum(new_fit_value)
    # Generate a sequence of random floating point numbers
    ms = []
    pop_len = len(pop)
    for i in range(pop_len):
        ms.append(random.random())
    # Sort the generated random sequence of floating point numbers
    ms.sort()
    # Roulette algorithm (the selected individual becomes the next round, the unselected individual is eliminated directly, and the selected individual is replaced)
    fitin = 0
    newin = 0
    newpop = pop
    while newin < pop_len:
        if (ms[newin] < new_fit_value[fitin]):
            newpop[newin] = pop[fitin]
            newin = newin + 1
        else:
            fitin = fitin + 1
    pop = newpop


# Sum up the fitness values
def sum(fit_value):
    total = 0
    for i in range(len(fit_value)):
        total += fit_value[i]
    return total


# Calculate cumulative probability
def cumsum(fit_value):
    temp = []
    for i in range(len(fit_value)):
        t = 0
        j = 0
        while (j <= i):
            t += fit_value[j]
            j = j + 1
        temp.append(t)
    for i in range(len(fit_value)):
```

```
            fit_value[i] = temp[i]


# Step 7: crossover
def crossover(pop, pc):  # 个体间交叉，实现基因交换
    poplen = len(pop)
    for i in range(poplen - 1):
        if (random.random() < pc):
            cpoint = random.randint(0, len(pop[0]))
            temp1 = []
            temp2 = []
            temp1.extend(pop[i][0: cpoint])
            temp1.extend(pop[i + 1][cpoint: len(pop[i])])
            temp2.extend(pop[i + 1][0: cpoint])
            temp2.extend(pop[i][cpoint: len(pop[i])])
            pop[i] = temp1
            pop[i + 1] = temp2


# Step 8: genetic mutations
def mutation(pop, pm):
    px = len(pop)
    py = len(pop[0])
    for i in range(px):
        if (random.random() < pm):
            mpoint = random.randint(0, py - 1)
            if (pop[i][mpoint] == 1):
                pop[i][mpoint] = 0
            else:
                pop[i][mpoint] = 1


def writeToFile(var, w_path):
    f = file(w_path, "a+")
    for item in var:
        f.write(str(item) + "\r\n")
    f.close()


def generAlgo(generations):
    pop = geneEncoding(pop_size, chrom_length)
    for i in range(generations):
        # print("第 " + str(i) + " 代开始繁殖......")
        obj_value = cal_obj_value(pop)  # Calculate the objective
```

```
function value
        # print(obj_value)
        fit_value = calfitvalue(obj_value)  # Calculate individual fitness value
        # print(fit_value)
        [best_individual, best_fit] = best(pop, fit_value)  # Pick the best individual and the best value of the function
        # print("best_individual: "+ str(best_individual))
        v1, v2, v3= b2d(best_individual)
        results.append([best_fit, v1, v2, v3])  # At each evolve, record the best results
        # print(str(best_individual) + " " + str(best_fit))
        selection(pop, fit_value)  # Natural selection, weeding out some of the less fit individuals
        crossover(pop, pc)  # crossover
        mutation(pop, pc)  # genetic mutation
    # print(results)
    results.sort()
    # wirte results to file
    writeToFile(results, "generation_" + str(generations) + ".txt")
    print(results[-1])
    # print(xgboostModel(100, 12))


if __name__ == '__main__':
    gen = [100, 200, 300, 400, 500]
    for g in gen:
        generAlgo(int(g))
```

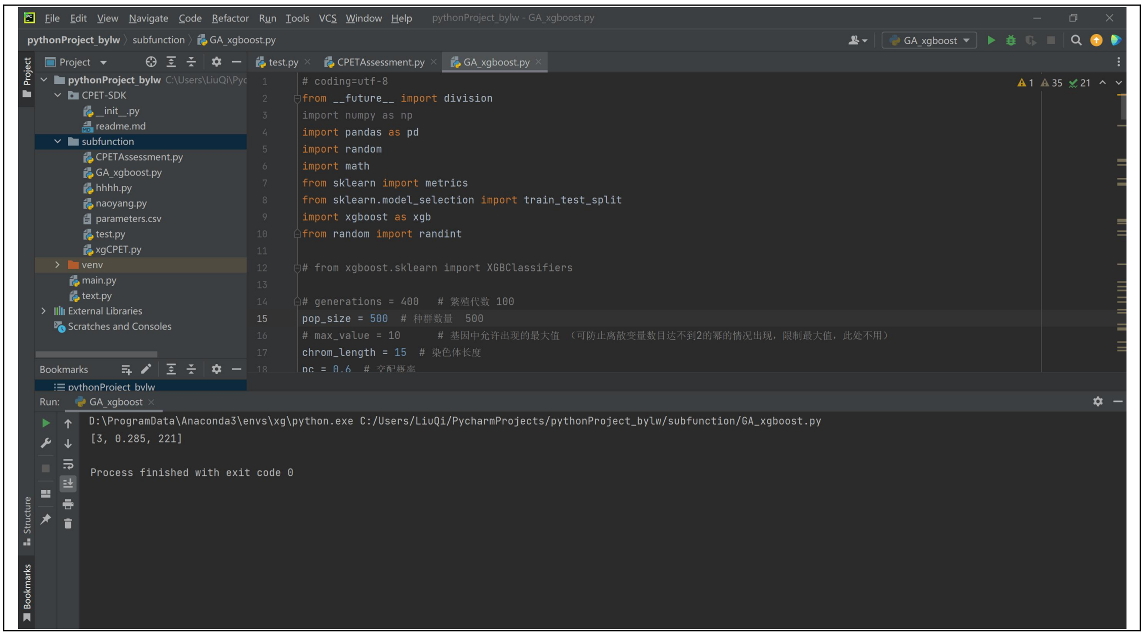

4. XGBOOST_SHAP(PYTHON)

```python
import xgboost as xgb
import pandas as pd
import numpy as np
import matplotlib.pyplot as plt
import shap
from sklearn.model_selection import train_test_split
from sklearn.metrics import accuracy_score
from sklearn.metrics import confusion_matrix
from sklearn.metrics import classification_report
from sklearn.metrics import precision_recall_fscore_support
from sklearn.metrics import f1_score

plt.style.use('seaborn')
data=pd.read_excel(r"D:\data.xlsx")
cols = ['x1', 'x2', 'x3', 'x4', 'x6', 'x7', 'x8', 'x9', 'x10', 'x11','x12','x14','x15','x16']
num=len(data)
X_train,X_test,y_train,y_test=train_test_split(data[cols],data['等级'],test_size=0.3,random_state=10)
model = xgb.XGBRegressor(max_depth=221, learning_rate=0.285, n_estimators=3)
model.fit(X_train, y_train)

## Interpretability analysis of the model
# get feature importance
plt.figure(figsize=(15, 5))
```

```python
plt.bar(range(len(cols)), model.feature_importances_)
plt.xticks(range(len(cols)), cols, rotation=-45, fontsize=14)
plt.title('Feature importance', fontsize=14)
plt.show()

explainer = shap.TreeExplainer(model)
shap_values = explainer.shap_values(X_train[cols])
shap.dependence_plot('x12',shap_values, X_train[cols]);


y_pred=model.predict(X_test)
predictions=[round(value) for value in y_pred]

# Calculate confusion matrix
confusion_matrix(y_test,predictions)

accuracy=accuracy_score(y_test,predictions)
# print('accu: %.2f%%' % (accuracy *100))
print(f1_score(y_test, predictions, average='macro'))
```

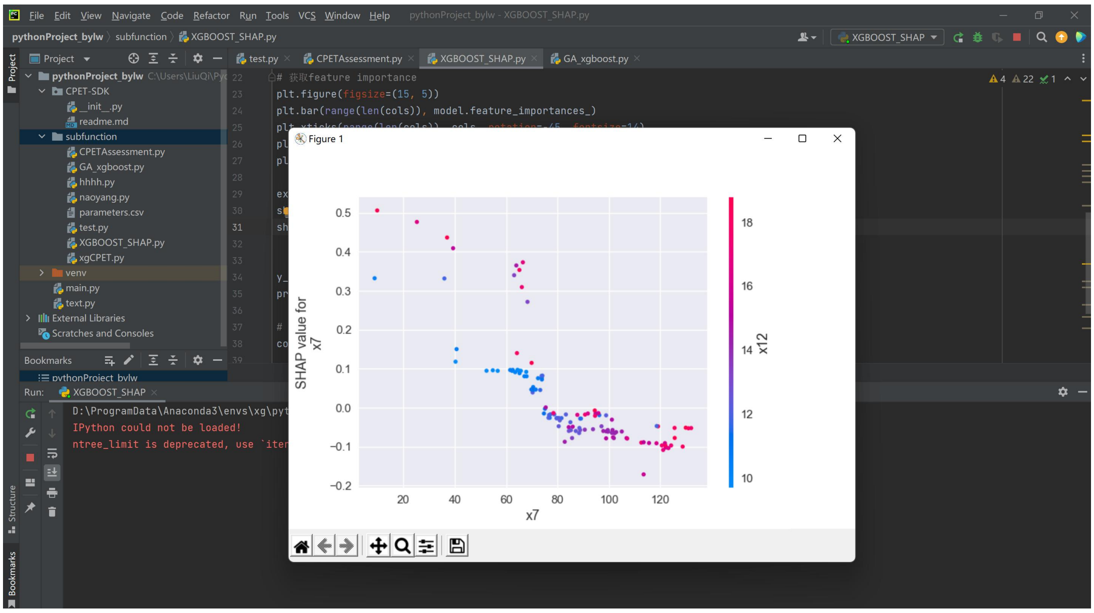

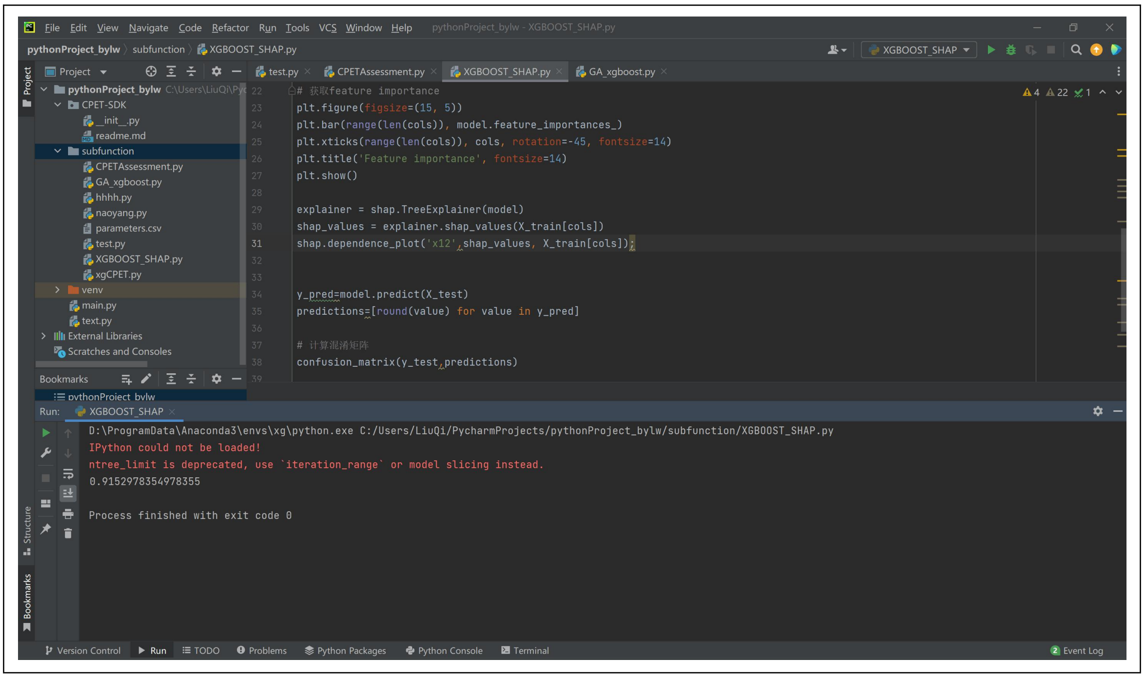

# 获取feature importance
plt.figure(figsize=(15, 5))
plt.bar(range(len(cols)), model.feature_importances_)
plt.xticks(range(len(cols)), cols, rotation=-45, fontsize=14)
plt.title('Feature importance', fontsize=14)
plt.show()
explainer = shap.TreeExplainer(model)
shap_values = explainer.shap_values(X_train[cols])
shap.dependence_plot('x12',shap_values, X_train[cols]);
y_pred=model.predict(X_test)
predictions=[round(value) for value in y_pred]
# 计算混淆矩阵
confusion_matrix(y_test,predictions)
D:\ProgramData\Anaconda3\envs\xg\python.exe C:/Users/LiuQi/PycharmProjects/pythonProject_bylw/subfunction/XGBOOST_SHAP.py
IPython could not be loaded!
ntree_limit is deprecated, use `iteration_range` or model slicing instead.
0.9152978354978355
Process finished with exit code 0
